# Thrombin and Plasmin Alter the Proteome of Neutrophil Extracellular Traps

**DOI:** 10.3389/fimmu.2018.01554

**Published:** 2018-07-09

**Authors:** Chun Hwee Lim, Sunil S. Adav, Siu Kwan Sze, Yeu Khai Choong, Rathi Saravanan, Artur Schmidtchen

**Affiliations:** ^1^Interdisciplinary Graduate School, NTU Institute for Health Technologies, Nanyang Technological University, Singapore, Singapore; ^2^Lee Kong Chian School of Medicine, Nanyang Technological University, Singapore, Singapore; ^3^School of Biological Sciences, Nanyang Technological University, Singapore, Singapore; ^4^Division of Dermatology and Venereology, Department of Clinical Sciences, Lund University, Lund, Sweden

**Keywords:** neutrophil extracellular traps, proteome, thrombin, plasmin, histones, neutrophil elastase

## Abstract

Neutrophil extracellular traps (NETs) consist of a decondensed DNA scaffold decorated with neutrophil-derived proteins. The proteome of NETs, or “NETome,” has been largely elucidated *in vitro*. However, components such as plasma and extracellular matrix proteins may affect the NETome under physiological conditions. Here, using a reductionistic approach, we explored the effects of two proteases active during injury and wounding, human thrombin and plasmin, on the NETome. Using high-resolution mass spectrometry, we identified a total of 164 proteins, including those previously not described in NETs. The serine proteases, particularly thrombin, were also found to interact with DNA and bound to NETs *in vitro*. Among the most abundant proteins were those identified previously, including histones, neutrophil elastase, and antimicrobial proteins. We observed reduced histone (H2B, H3, and H4) and neutrophil elastase levels upon the addition of the two proteases. Analyses of NET-derived tryptic peptides identified subtle changes upon protease treatments. Our results provide evidence that exogenous proteases, present during wounding and inflammation, influence the NETome. Taken together, regulation of NETs and their proteins under different physiological conditions may affect their roles in infection, inflammation, and the host response.

## Introduction

In 2004, Brinkmann et al. observed a novel extracellular structure formed by neutrophils which they coined neutrophil extracellular traps (NETs). They are made up of decondensed DNA as the structural backbone for various neutrophil-derived proteins such as histones and neutrophil elastase (ELANE) (1). NETs are exuded in response to stimuli such as microorganisms, chemical derivatives including phorbol 12-myristate 13-acetate (PMA) and calcium ionophores, lipopolysaccharides, and even activated endothelial cells. Studies have shown that NETs exert antimicrobial effects on various microorganisms including bacteria, fungi, and parasites (2). *In vivo*, they have been identified in several inflammatory conditions and are also implicated in the pathogenesis of autoimmune diseases (3).

Neutrophil extracellular trap-associated proteins, particularly enzymes, have been described to be biologically active (4–6). Hence, it is of interest to study the proteome of NETs to gain insights into its functions. Two separate proteomics studies identified a total of 24 (7) and 29 (6) NET-associated proteins predominated by histones, ELANE, and several antimicrobial proteins or peptides. In the experimental set-ups of these studies, however, only neutrophil-derived proteins were reported (6, 7). Separately, non-proteomic-based studies have described the binding of the complement component C1q to NETs (8). Therefore, the full repertoire of NETs’ proteins has not yet been elucidated, especially under physiological and inflammatory conditions.

Neutrophil extracellular traps have been identified during wounding, where other proteins are also variably regulated (9–12). For example, thrombin initializes hemostasis by converting fibrinogen to fibrin, which in turn is proteolyzed by plasmin during the later fibrinolytic phase of wound healing (13). With this as background, we hypothesized that these two serine proteases may alter the proteome of NETs. Using high-resolution liquid chromatography-mass spectrometry (LC-MS/MS), molecular biology, and bioinformatics tools, we therefore explored changes of NET-associated proteins after addition of thrombin or plasmin. We observed differences in the abundances of multiple NET-proteins, demonstrating that the NETome is dynamically regulated and shows qualitative and quantitative changes depending on the environment.

## Materials and Methods

### Materials

Sodium citrate-containing tubes used for blood collection were from Becton-Dickinson, USA. Polymorphprep was purchased from Axis-Shield, Scotland. The Erythrocyte Lysis Buffer was from eBioscience, USA. The fluorescently labeled antibodies used for flow cytometry—Brilliant Violet 510 (BV510)-anti-CD45 (clone HI30) and fluorescein isothiocyanate-anti-CD66b (clone G10F5)—were from BioLegend, USA. Rose Park Memorial Institute (RPMI)-1640 cell culture medium without phenol red, the live-dead stain 4′,6-diamidino-2-phenylindole (DAPI), DNA endonuclease I (DNase I), Pierce Protease Inhibitor Mini Tablets, Pierce Silver Stain Kit, Pierce BCA (bicinchionic acid) Protein Assay Kit, Supersignal West Dura Extended Duration Substrate, Supersignal West Femto Maximum Sensitivity Substrate, and SYBR Safe were from Thermo Fisher Scientific, USA. PMA, poly-l-lysine, and Fluoroshield with DAPI mounting medium were bought from Sigma-Aldrich, USA. Purified thrombin and plasmin were from Innovative Research, USA. Sequencing grade modified trypsin was from Promega, USA. Recombinant histones H2B, H3.3, and H4 were from New England Biolabs, USA. Purified ELANE was from Merck, Germany. The 4–20% polyacrylamide Mini-PROTEAN Tris–Glycine precast gels and Immun-Blot polyvinylidene fluoride (PVDF) membrane were from Bio-Rad, USA. The E.Z.N.A. DNA/RNA Isolation Kit was from Omega Bio-Tek, USA. The rabbit polyclonal antibody recognizing the thrombin C-terminal peptide (α-VFR17; VFRLKKWIQKVIDQFGE) was from Innovagen AB, Sweden. Antibodies from Abcam, USA include—rabbit polyclonal anti-H2B (α-H2B), anti-H3 (α-H3), anti-H4 (α-H4), and anti-ELANE (α-ELANE) antibodies; goat polyclonal anti-plasminogen antibody (α-PLG); horse radish peroxidase (HRP)-conjugated donkey polyclonal anti-goat IgG antibody (HRP-α-goat); Alexa-Fluor (AF)-568 goat polyclonal anti-rabbit IgG antibody (AF568-α-rabbit); and AF488 donkey polyclonal anti-goat IgG antibody (AF488-α-goat). The HRP-conjugated goat polyclonal anti-rabbit IgG antibody (HRP-α-rabbit) was from Agilent, USA. For blocking of western blots or immunofluorescence staining, either 3% milk (w/v), 3 or 5% bovine serum albumin (BSA; w/v) was dissolved in TBS-T (Tris-buffered saline with 0.1% v/v Tween-20) and used, as indicated.

### Ethics Statement

Human whole blood samples were obtained from participating subjects who gave written informed consent. The process of blood sample collection and methods used thereafter were performed in accordance with the guidelines and regulation recommended and approved by the Nanyang Technological University Singapore’s Institutional Review Board (IRB-2014-10-041).

### Polymorphonucleated Cells (PMNs) Isolation

Whole blood obtained in sodium citrate-containing tubes by venipuncture was used for PMNs isolation with Polymorphprep according to the manufacturer’s instructions. Briefly, approximately 1 part of blood was layered onto 1 part of Polymorphoprep before centrifugation at 500 × *g* for 30 min at 25°C without brakes. The separated layer containing PMNs was then harvested, treated with Erythrocyte Lysis Buffer, washed, and resuspended in RPMI-1640. The purity of isolated PMNs was determined by flow cytometry (LSR-Fortessa-X20; Becton-Dickinson, USA), and the acquired data were analyzed with Flow Jo (Tree Star, USA). PMN isolates at >97% were used, as defined by the percentage of CD66b+ cells over live CD45+ cells.

### NET Induction

**Figure 1:**
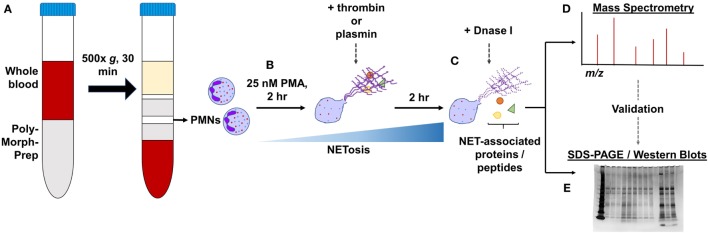
General experimental workflow. **(A)** Polymorphonucleated cells (PMNs) were isolated from three independent human whole blood samples by density centrifugation and (B) treated with 25 nM PMA for 2 h to induce neutrophil extracellular traps (NETs). PMNs were left untreated or supplemented with 1 µM thrombin or plasmin and incubated for another 2 h. (C) After gently washing the wells, they were further treated with 20 U/ml DNase I or with Rose Park Memorial Institute only for 1 h at 37°C to digest the DNA scaffold. The proteins extracted were then processed for (D) LC-MS/MS or (E) in some cases, subjected to validation by sodium dodecyl sulfate-polyacrylamide gel electrophoresis (SDS-PAGE)/western blotting.

Isolated PMNs (1 × 10^6^ cells/ml; 2 million cells) were seeded in 6-well plates and treated with 25 nM PMA for a total of 4 h at 37°C to induce NET formation. At the 2-h time-point, 1 µM thrombin or plasmin was added, whereas the control wells were left as they were. The wells were not washed during this period. Next, the wells were washed gently three times with prewarmed RPMI before further treatments with 20 U/ml DNase I in RPMI (+DNase) or RPMI alone (−DNase) for 1 h at 37°C. The supernatants in the respective wells were then carefully aspirated, added with protease inhibitor, and processed for mass spectrometry or sodium dodecyl sulfate-polyacrylamide gel electrophoresis (SDS-PAGE) (Figure 1). A total of three biological replicates from three human subjects were prepared. The amounts of proteins in each sample were estimated using the Pierce BCA Protein Assay Kit.

### LC-MS/MS and Data Analysis

To prepare the samples for LC-MS/MS, 40 µg of the NET-proteins was subjected to SDS-PAGE (5% polyacrylamide stacking gel with 12% polyacrylamide separating gel). Electrophoresis was first run at 80 V for 20 min, then at 100 V for another 20 min. Next, each sample lane was separately sliced and cut into pieces (approximately 1 mm^2^), washed with buffer (75% acetonitrile containing 25 mM ammonium bicarbonate), and de-stained completely. Then, these gel pieces were reduced with 10 mM dithiothreitol and alkylated with 55 mM iodoacetamide in the dark. They were later dehydrated with 100% acetonitrile and subjected to sequencing grade modified trypsin digestion at 37°C, overnight. The peptides were extracted using 50% acetonitrile/5% acetic acid. Finally, the extracted peptides were desalted and vacuum centrifuged to dryness.

For LC-MS/MS, the peptides were reconstituted in 0.1% formic acid (FA). Three biological replicates and their technical duplicates were separated and analyzed in a LC-MS/MS system comprising a Dionex Ultimate 3000 RSLC nano-HPLC system, coupled with an online Q-Exactive mass spectrometer (Thermo Fisher Scientific, USA). Five microliters of each sample were injected into an acclaim peptide trap column *via* the auto-sampler of the Dionex RSLC nano-HPLC system. The flow rate was maintained at 300 nl/ml. The mobile phase A (0.1% FA in 5% acetonitrile) and mobile phase B (0.1% FA in acetonitrile) were used to establish a 60-min gradient. Peptides were analyzed on a Dionex EASY-spray column (PepMap^®^ C18, 3 μm, 100 A) using an EASY nanospray source. The electrospray potential was set at 1.5 kV. Full MS scan in the range of 350–1,600 *m*/*z* was acquired at a resolution of 70,000 at *m*/*z* 200, with a maximum ion accumulation time of 100 ms. Dynamic exclusion was set to 30 s. Resolution for MS/MS spectra was set to 35,000 at *m*/*z* 200. The AGC setting was 1E6 for the full MS scan and 2E5 for the MS2 scan. The 10 most intense ions above 1,000 count threshold were chosen for higher energy collision dissociation (HCD) fragmentation. The maximum ion accumulation time was 120 ms. An isolation width of 2 Da was used for the MS2 scan. Single and unassigned charged ions were excluded from MS/MS. For HCD, normalized collision energy was set to 28. The underfill ratio was defined as 0.1%.

For data analysis, the raw data files were converted into the Mascot generic file format using Proteome Discoverer version 1.4 (Thermo Electron, Germany) with the MS2 spectrum processor for the de-isotoping of the MS/MS spectra. The concatenated target-decoy UniProt human database (total sequences 92867, total residues 36953354, downloaded on 25 July 2016) was used for data searches. Data search was performed using in-house Mascot server (version 2.4.1, Matrix Science, USA). Static peptide modification was carbamidomethylation of cysteine residues (+57.021 Da), and the dynamic peptide modifications were oxidation of methionine residues (+15.995 Da) and deamidation of asparagine and glutamine residues (+0.984 Da). Two missed cleavage sites of trypsin and mass tolerances of 10 parts per million for peptide precursors were allowed. A mass tolerance of 0.8 Da was set for fragmented ions in Mascot searches. Quantification was performed using exponentially modified protein abundance index (emPAI) scores reported by Mascot search engine which is based on equations as stated below (14):

$$\text{PAI}=\frac{N_{\text{obsd}}}{N_{\text{obsbl}}}$$

where *N*_obsd_ and *N*_obsbl_ are the number of observed peptides per protein and the number of observable peptides per protein, respectively. The emPAI is defined as follows:

$$\text{emPAI}=10^{\text{PAI}}-1$$

Subsequently, the identified proteins dataset was exported into Microsoft Excel for further analysis. The false discovery rate (FDR) was calculated using in-house build program as 2 × Md/(Md + Mt), where Md represents the number of decoy matches, and Mt is the number of target matches. Proteins with FDR ≤ 1 were considered for further analysis. To identify NET-associated proteins, we adopted the workflow to eliminate proteins that were constitutively released from our culture conditions without DNase I (Figure S1 in Supplementary Material). Finally, the hierarchical clustering of true NET-associated protein abundances was generated using GenePattern 2.0 (15).

### SDS-PAGE and Protein Detection

Equal amounts of NET-proteins, where indicated, were loaded onto 4–20% polyacrylamide gel for SDS-PAGE. Then, the gel was used for either silver staining with the Pierce Silver Stain Kit (according to the manufacturer’s instructions) or transferred onto PVDF membranes for western blotting. For the detection of thrombin and plasmin, the blots were first blocked with 5 or 3% milk/TBS-T. Then, the blots were incubated with α-VFR17 (1:5,000 in 5% milk/TBS-T) and α-PLG (1:1,000 in 3% milk/TBS-T). Finally, HRP-α-rabbit antibody (1:10,000 in 5% milk/TBS-T) and HRP-α-goat antibody (1:10,000 in 3% milk/TBS-T) were used to detect the respective primary antibodies. For the detection of histones, the blots were first blocked with 5% BSA/TBS-T. Then, they were incubated with α-H2B (1:1,000), α-H3 (1:1,000), and α-H4 (1:5,000) diluted in 5% BSA/TBS-T. The primary antibodies were than detected with HRP-α-rabbit (1:10,000) diluted in 1% BSA/TBS-T. For the detection of neutrophil elastase, the blot was first blocked with 3% milk/TBS-T and probed with α-ELANE (1:1,000) diluted in 3% milk/TBS-T. Finally, α-ELANE was detected with HRP-α-rabbit antibody (1:10,000) diluted in 3% milk/TBS-T. All the blots were developed using the enhanced chemiluminescence method with either the Supersignal West Dura or Femto Substrate and visualized with the ChemiDoc Imaging System (Bio-Rad, USA).

### DNA Gel Mobility Shift Assay

DNA was purified from isolated PMNs using the E.Z.N.A. DNA/RNA Isolation Kit according to the manufacturer’s instructions. Then, 20 µg/ml of the DNA was incubated alone or with 1.0, 2.0, 4.0, 8.0, or 12.0 µM of thrombin or plasmin in RPMI for 1 h at 37°C. This was followed by gel electrophoresis on a 0.5% agarose (w/v) gel in Tris acetate-EDTA buffer with SYBR Safe which was then visualized with the ChemiDoc Imaging System (Bio-Rad, USA).

### Immunofluorescence

Polymorphonucleated cells (5 × 10^5^ cells/ml; 100,000 cells) were first seeded onto 0.1 mg/ml poly-l-lysine-coated coverslips in 24-well plates before NET induction with PMA and treatment with 1 µM of thrombin or plasmin as described above. After the complete 4-h procedure, the wells were gently washed two times with prewarmed RPMI, fixed with 4% PFA, and washed another two times with TBS-T. All samples were blocked with 3% BSA/TBS-T before incubation with the respective primary and fluorescently labeled secondary antibodies diluted in 3% BSA/TBS-T. The dilutions used were—α-H2B (1:350), α-VFR17 (1:1,000), α-plasminogen (1:500), AF568-goat polyclonal α-rabbit IgG (1:1,000), and AF488-donkey polyclonal α-goat IgG (1:1,000). Finally, the coverslips were mounted using the Fluoroshield with DAPI mounting medium.

### Peptigrams

Peptigrams were generated (available at http://bioware.ucd.ie/peptigram/) and used to compare the tryptic peptides detected with modifications. Each tryptic peptide detected within the sample with peptide scores of more than 20 were summed (if more than 1 of the same peptide was detected) to give an overall view of the peptide’s abundance. The data were then processed for peptides corresponding to their respective proteins as described (16).

### Statistical Analysis

Statistical analyses were performed on the proteins’ emPAI scores using the Prism software version 7.0 (GraphPad, USA). One-way ANOVA with Dunnett’s correction for multiple comparison was applied unless otherwise indicated. Data was presented as mean (SD). Statistical significance based on *p* values were—^#^*p* < 0.1 (not significant), *p < 0.05, **p < 0.01, ***p < 0.001, and ****p < 0.0001.

## Results

### Identification of NET-Associated Proteins

**Figure 2:**
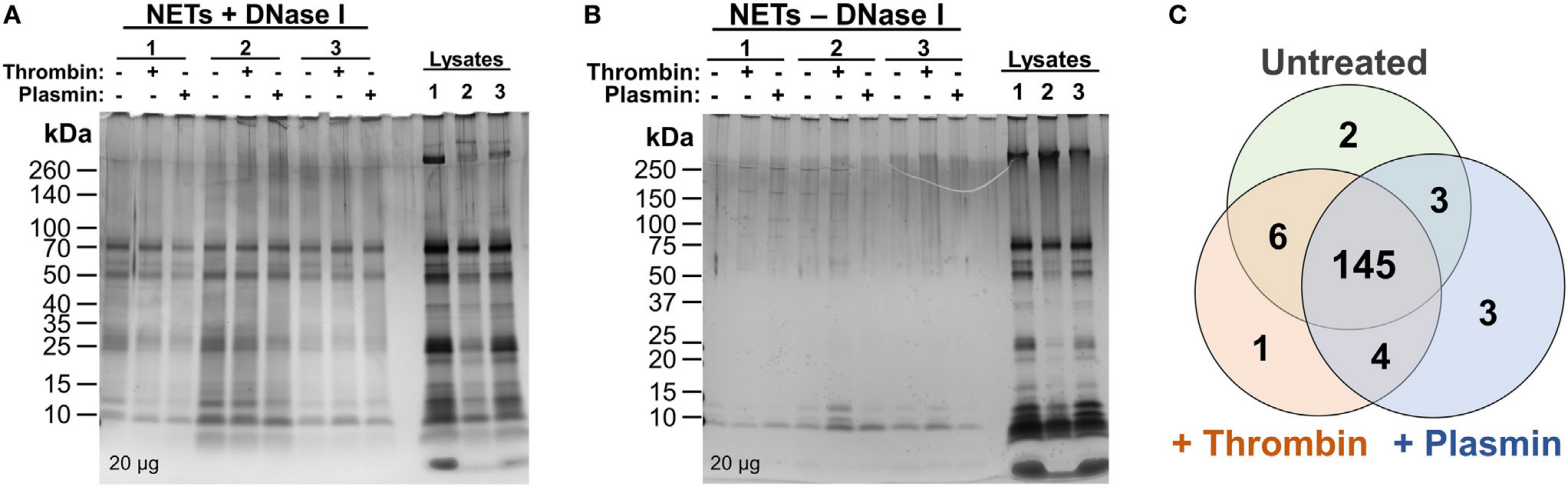
Silver stains and initial comparison of neutrophil extracellular trap (NET)-proteins. **(A,B)** Proteins extracted from NETs were quantified and loaded (20 µg) onto 4–20% Tris–Glycine gradient gels. Silver staining was then performed—**(A)** DNase I-treated samples and **(B)** non-DNase I-treated samples. **(C)** Proteins identified as NET-bound (as illustrated in Figure S1 in Supplementary Material) from untreated, 1 µM thrombin-, or 1 µM plasmin-treated samples were compared using Venn diagram.

Previous reports of the proteome of NETs described the identification and quantification of NET-associated proteins from purified neutrophils only and without additional, exogenous components (6, 7). Here, we aimed to investigate whether the coagulation proteases—thrombin and plasmin—have any effects on the NETome. The experiments were performed in the absence of serum to exclude potential influences from other serum proteases. PMA was used as the NET-inducing agent to provide a basal, standardized, NETome for comparison. Following NET induction procedures as described in Figure 1, we first analyzed whether DNase I was able to release NET-associated proteins by SDS-PAGE followed by silver staining (Figures 2A,B). We observed significantly more proteins in DNase I-treated samples (Figure 2A) when compared with the non-DNase I-treated material (Figure 2B). This indicated that most of these proteins were released upon DNase I treatment and thus, NET-bound.

Next, the samples were sent for high-resolution LC-MS/MS to identify the proteins present. To correctly identify the NET-associated proteins, we adopted a data processing workflow to ensure that proteins non-specifically released into the medium during DNase I treatment were screened out (i.e., from cell membranes or those attached to the wells) (Figure S1 in Supplementary Material). Protein correlations of the samples between donors were also performed (>0.79, Figure S2 in Supplementary Material) and demonstrated good data reproducibility for the experiments.

Consequently, 164 NET-associated proteins were identified (Figure 2C), a number higher than previously reported (6, 7). The higher ranked proteins identified with high protein scores were congruent with those previously reported as observed in Table 1. In addition, we identified proteins not described to be present on NETs previously, such as the serine protease inhibitor (Serpin)-B1, lipocalin-2, and complement C3 (Table 1). Peptides corresponding to the antimicrobial proteins cathelicidin and S100A12 were also detected; however, they were excluded during data analysis due to the FDR ≤ 1 applied.

**Table 1:** Comparison of neutrophil extracellular trap-associated proteins identified.

Function	Protein	Gene	Protein rank	Accession no.	Urban et al. (7)	O’Donoghue et al. (6)	This study
**Nuclear localization**	Histone H1	*H1*	99	P10421		Y	Y
			93	P16403			
			145	P16401			
	Histone H2A	*H2A*	11	Q16777	Y	Y	Y
	Histone H2B	*H2B*	8	Q5QNW6	Y	Y	Y
			4	Q16778			
	Histone H3	*H3*	16	P84243	Y	Y	Y
			21	Q5TEC6			
	Histone H4	*H4*	1	P62805	Y	Y	Y
	Myeloid cell nuclear differentiation antigen	*MNDA*	32	P41218	Y	Y	Y

**Proteolytic enzymes**	Neutrophil elastase	*ELANE*	12	P08246	Y	Y	Y
	Cathepsin G	*CTSG*	9	P08311	Y	Y	Y
	Leukocyte proteinase 3/myeloblastin	*PRTN3*	43	P24158	Y	Y	Y
	Lipocalin-2	*LCN2*	20	P80188			Y

**Glycolytic enzymes**	Alpha-enolase	*ENO1*	84	P06733	Y	Y	Y
	Transketolase	*TKT*	156	P29401	Y	Y	Y

**Antimicrobial**	Lactotransferrin	*LTF*	2	P02788	Y	Y	Y
	Azurocidin	*AZU*	10	P20160	Y	Y	Y
	Lysozyme C	*LYZ*	26	P61626	Y	Y	Y
	Neutrophil defensin 1 or 3	*DEFA1 or DEFA3*	18	P59665 or P59666	Y	Y	Y
	S100A8	*S100A8*	19	P05109	Y	Y	Y
	S100A9	*S100A9*	7	P06702	Y	Y	Y
	S100A12	*S100A12*	–	P80511	Y	Y	
	Cathelicidin LL37	*CAMP*	–	P49913		Y	
	Eosinophil cationic protein	*RNASE3*	75	P12724			Y

**Cytoskeleton**	Actin	*ACTB*	14	P60709	Y	Y	Y
	Myosin-9	*MYH9*	154	P35579	Y	Y	Y^#^
	Alpha-actinin-1	*ACTN1*	150	P12814	Y		Y^#^
	Alpha-actinin-4	*ACTN4*	135	O43707	Y		Y
	Plastin-2	*LCP1*	104	P13796	Y	Y	Y
	Cytokeratin-10	*KRT10*	17	P13645	Y		Y

**Oxidative response**	Myeloperoxidase	*MPO*	3	P05164	Y	Y	Y
	Catalase	*CAT*	116	P04040	Y		Y

**Protease inhibitors**	SerpinB1	*SERPINB1*	27	P30740			Y
	SerpinA1	*SERPINA1*	106	P01009			Y

**Other inflammatory responses**	Complement C3	*C3*	114	P01024			Y
	Serotransferrin	*TF*	42	P02787			Y

*Studies from Urban et al. (7) and O’Donoghue et al. (6) were compared against the present study*.

*Y, identified in the studies; Y^#^, identified in plasmin-treated samples only; red box, not identified in the study; blue box, newly identified or previously identified in only one of the two studies*.

### Thrombin and Plasmin Can Bind to NETs

**Figure 3:**
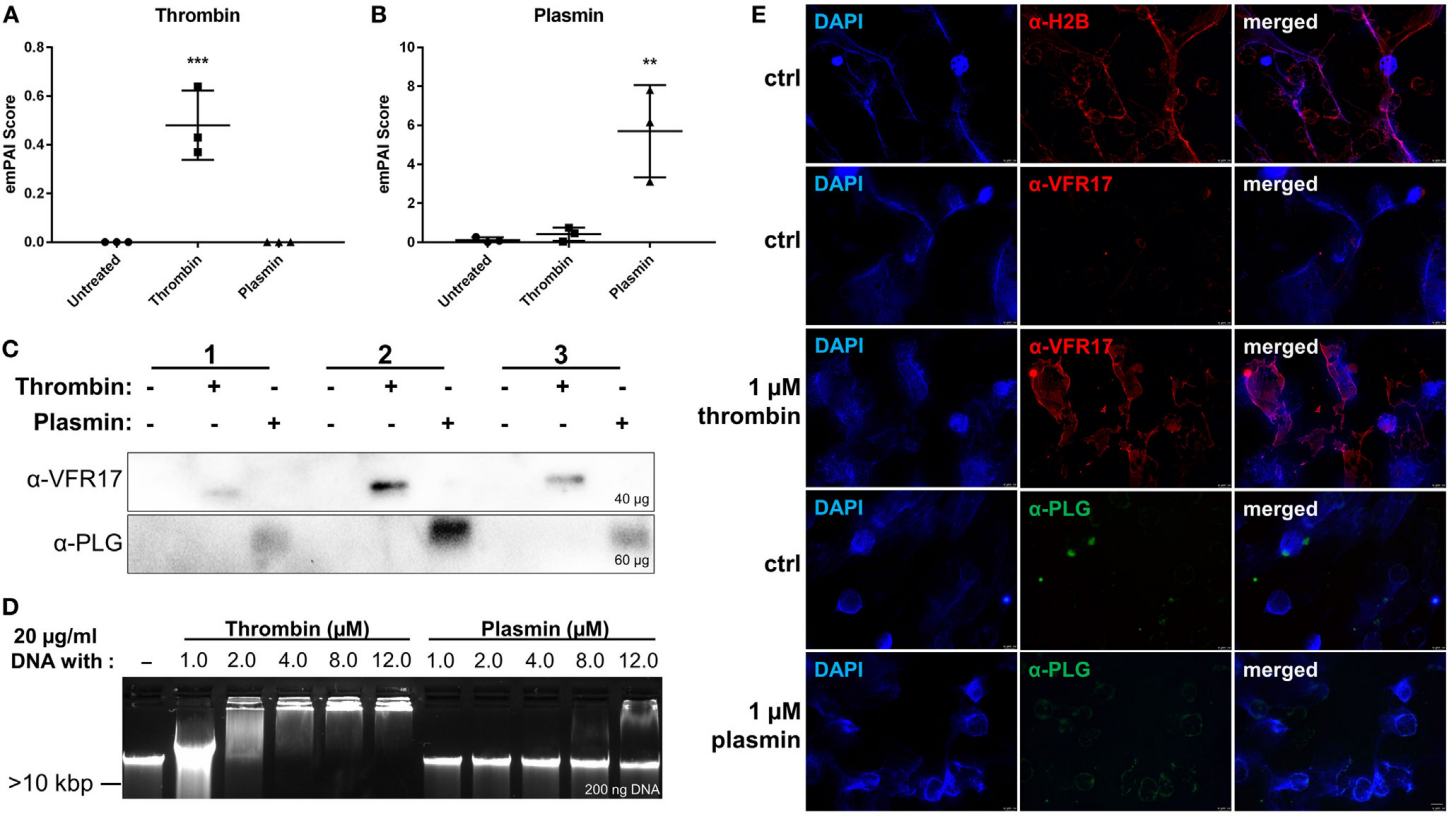
Analysis of neutrophil extracellular trap/DNA-binding of thrombin and plasmin. The raw exponentially modified protein abundance index (emPAI) scores from + DNase samples of **(A)** thrombin and **(B)** plasmin were compared across the treatment conditions (*n* = 3). **(C)** Western blot analyses on +DNase samples for α-VRF17 (~37 kDa; *upper panel*) and α-PLG (~40 kDa; *lower panel*). Amounts of proteins loaded were as indicated in the figure. **(D)** DNA (20 µg/ml) was either incubated alone or with 1.0, 2.0, 4.0, 8.0, or 12.0 µM of thrombin or plasmin in Rose Park Memorial Institute for 1 h at 37°C. Then, 200 ng of DNA was loaded onto 0.5% agarose gel for gel electrophoresis. **(E)** Polymorphonucleated cells were treated with PMA only (*ctrl*) for 4 h at 37°C or added with 1 µM thrombin or plasmin after 2 h with PMA. *First row*: α-H2B (red); *second* and *third row*: α-VFR17 (red); *fourth* and *fifth row*: α-PLG (green). Scale bar: 10 µm.

The two S1 peptidases thrombin and plasmin have been reported to interact with DNA aptamers (17) and short oligos (18). In the initial experiments, we explored whether these proteins can bind to the DNA of NETs. Notably, based on the mass spectrometry data obtained, we detected the presence of thrombin and plasmin in the NET samples treated with the respective enzyme (Figures 3A,B). Moreover, the presence of the two proteases was further validated by western blotting, where antibodies specific for thrombin (~37 kDa) and plasmin (~40 kDa), detected these proteins in the respective NET samples under reducing conditions (Figure 3C). To investigate whether the two proteases interact with DNA, thrombin and plasmin were incubated with purified PMN DNA and analyzed by a gel mobility shift assay. We observed reduced band mobility after addition of 1.0 µM thrombin and complete DNA retardation at or above 4.0 µM thrombin. For plasmin, however, reduced band mobility was observed at 8.0 µM plasmin and above, indicating that plasmin showed a weaker interaction with DNA in comparison with thrombin (Figure 3D). Immunofluorescence microscopy analysis showed that thrombin, but not plasmin (both at 1 µM), co-localized with NETs (Figure 3E). In contrast to thrombin, which was found to associate with NET-like structures, plasmin was bound to the cell membrane and/or cell debris (Figure 3E lower panel), compatible with previous studies showing that plasmin binds to PMN surfaces (19). Notably, when plasmin levels from plasmin-treated NETs were compared with controls, there was still an observable increase in plasmin abundance following DNase I treatment, indicating that plasmin was NET-bound and released upon DNase I treatment (Figure S3B in Supplementary Material). Taken together, the data show that particularly thrombin can bind to NETs of PMNs.

### Effects of Thrombin and Plasmin on NET-Associated Histones

**Figure 4:**
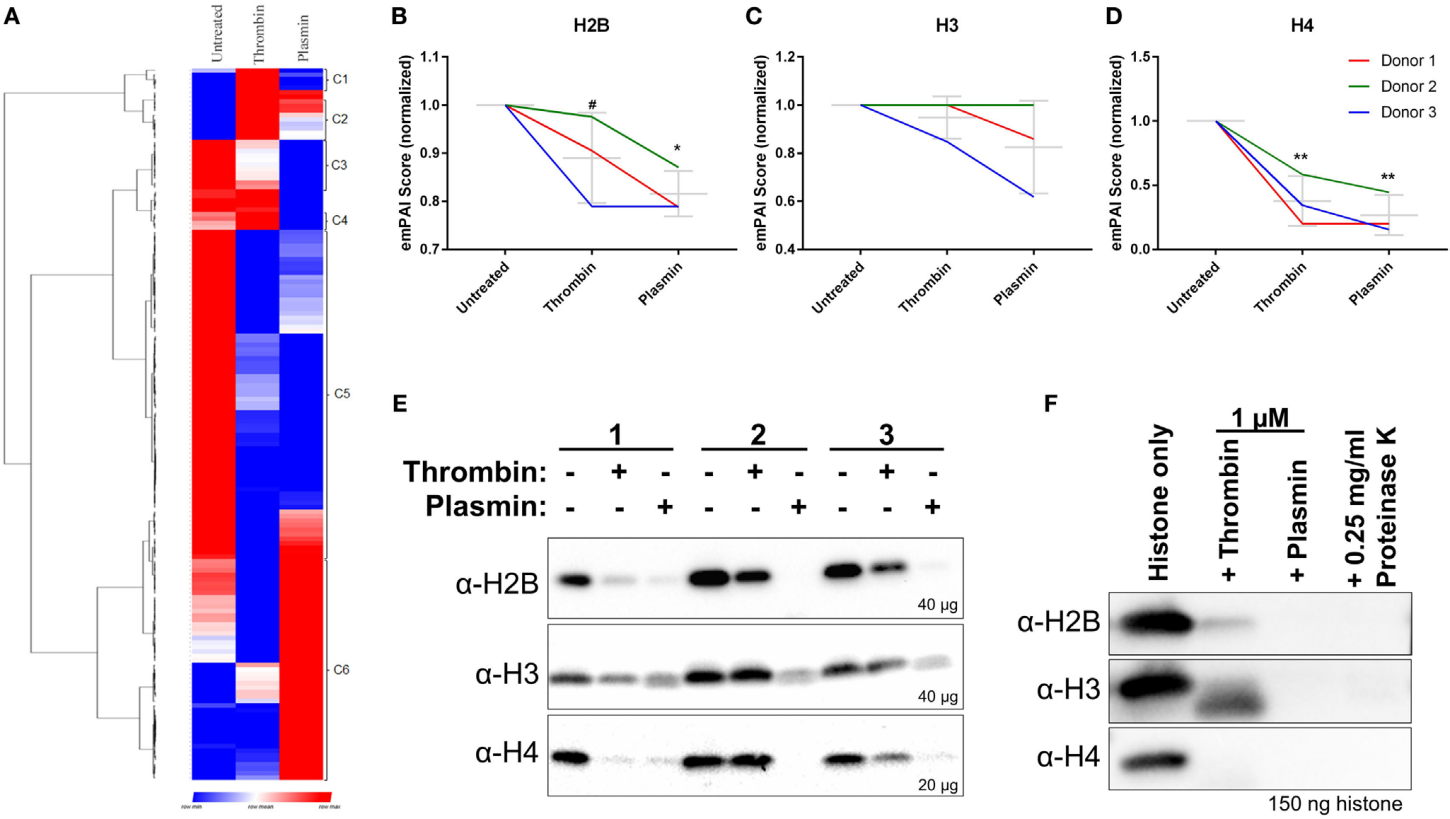
Reduced major histones on neutrophil extracellular traps (NETs) by thrombin and plasmin. **(A)** Hierarchical clustering of identified NET-associated proteins following data processing in Figure S1 in Supplementary Material. Enriched proteins are displayed in *red*, lowered proteins, are displayed in *blue* and the intermediate values are displayed in the shades of *red* and *blue*. **(B–D)** The histones’ exponentially modified protein abundance index (emPAI) scores from thrombin- and plasmin-treated samples were normalized against the respective donor’s untreated control. Donor-matched samples are presented (*n* = 3). **(B)** emPAI scores for histone H2B subtypes E and F for each treatment were summed then normalized against the untreated control. **(C)** emPAI scores for histone H3 and variant H3.3 for each treatment were summed then normalized against the untreated control. **(D)** Normalized histone H4 emPAI scores. **(E)** Western blot analyses on +DNase samples using α-H2B (~12 kDa; *upper panel*), α-H3 (~11 kDa; *middle panel*), and α-H4 (~9 kDa; *lower panel*) antibodies. Amounts of proteins loaded for each sample were as indicated. **(F)** Recombinant histones H2B, H3.3, and H4 (2 µM) were treated with 1 µM thrombin, 1 µM plasmin, or 0.25 mg/ml proteinase K for 2 h at 37°C before sodium dodecyl sulfate-polyacrylamide gel electrophoresis with 150 ng of the respective histones and western blotting. Antibodies used were α-H2B (~15 kDa; *upper panel*), α-H3 (~15 kDa; *middle panel*), and α-H4 (~13 kDa; *lower panel*).

In addition to the identification of new NET-associated proteins, we compared the protein abundances across the three treatment conditions by hierarchical clustering. The proteins were classified into eight clusters—proteins enriched in untreated samples (*cluster C3* and *C5*), thrombin-treated samples (*cluster C1, C2*, and *C4*), and plasmin-treated samples (*cluster C6*) (Figure 4A; Figure S4 in Supplementary Material). The clusters *C3* and *C5* consist of many classically NET-associated proteins such as histones, ELANE, and some antimicrobial proteins (Figure S4A in Supplementary Material). As histones are significant components of NETs (7), three histones—H2B, H3, and H4 (Figures 4B–D)—were selected for analysis by western blotting. The results show that these NET-associated histones were reduced in the presence of thrombin and plasmin which corresponded to the obtained mass spectrometry data (Figure 4E vs. Figures 4B–D).

As thrombin and plasmin are proteases, we explored whether their proteolytic activities resulted in the observed reduction of histone levels. To address this, the recombinantly produced histones H2B, H3.3, and H4 were incubated with thrombin or plasmin. We observed that the three types of histones were indeed degraded in the presence of the two proteases (Figure 4F), which was in agreement with the immunoblot results (Figure 4E). This suggests that the thrombin- and plasmin-mediated reduction of the studied NET-associated histones is due to proteolysis.

**Figure 5:**
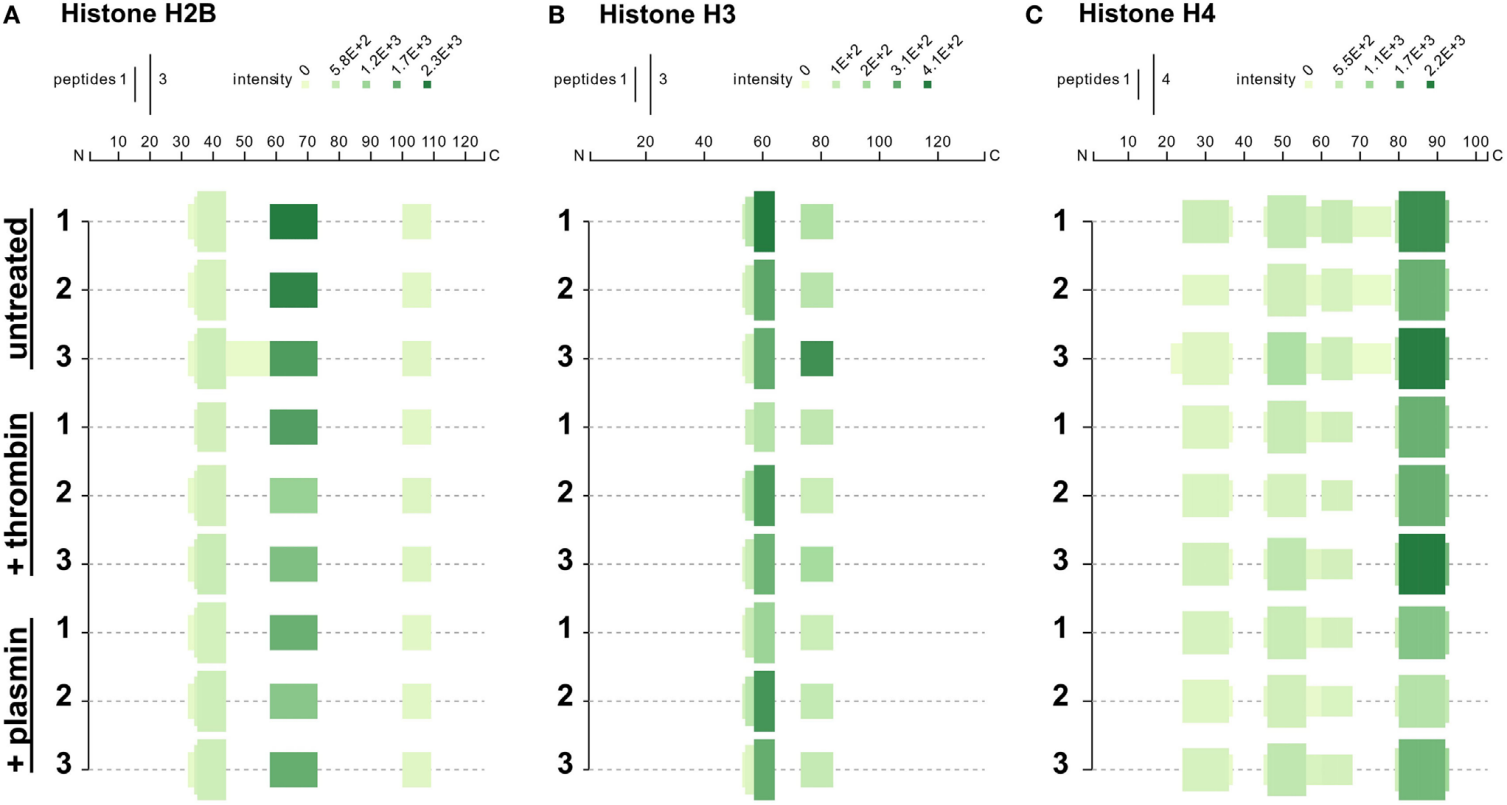
Peptigrams for histones H2B, H3.3, and H4. Peptigrams were generated using the sum of the respective peptide scores corresponding to **(A)** histone H2B subtype F (Q5QNW6), **(B)** histone H3 (Q5TEC6), and **(C)** histone H4 (P62805).

We next investigated whether treatment with thrombin and plasmin may alter the patterns of histone peptide fragments detected by mass spectrometry. To this end, the unique tryptic peptides identified by mass spectrometry were aligned to the respective full-length histones and presented in peptigrams (16) (Figure 5). Corresponding to the results presented in Figure 4, we observed that the peptide intensities of the NET-associated histones were reduced after treatment with thrombin or plasmin. However, there were no major differences regarding the origins of the peptide sequences detected for histones H2B and H3 (Figures 5A,B). For histone H4 on the other hand, the peptide from residues 61–78 (VFLENVIRDAVTYTEHAK; 61V–78K) was not detected in all thrombin- and plasmin-treated samples (Figure 5C), suggesting that this region was affected by thrombin and plasmin. Taken together, in addition to providing useful information on the peptide sequences detected and their abundances, these results also reflect the specificity, sensitivity, and reproducibility of our experimental workflow.

### Effects of Thrombin and Plasmin on NET-Associated Neutrophil Elastase

**Figure 6:**
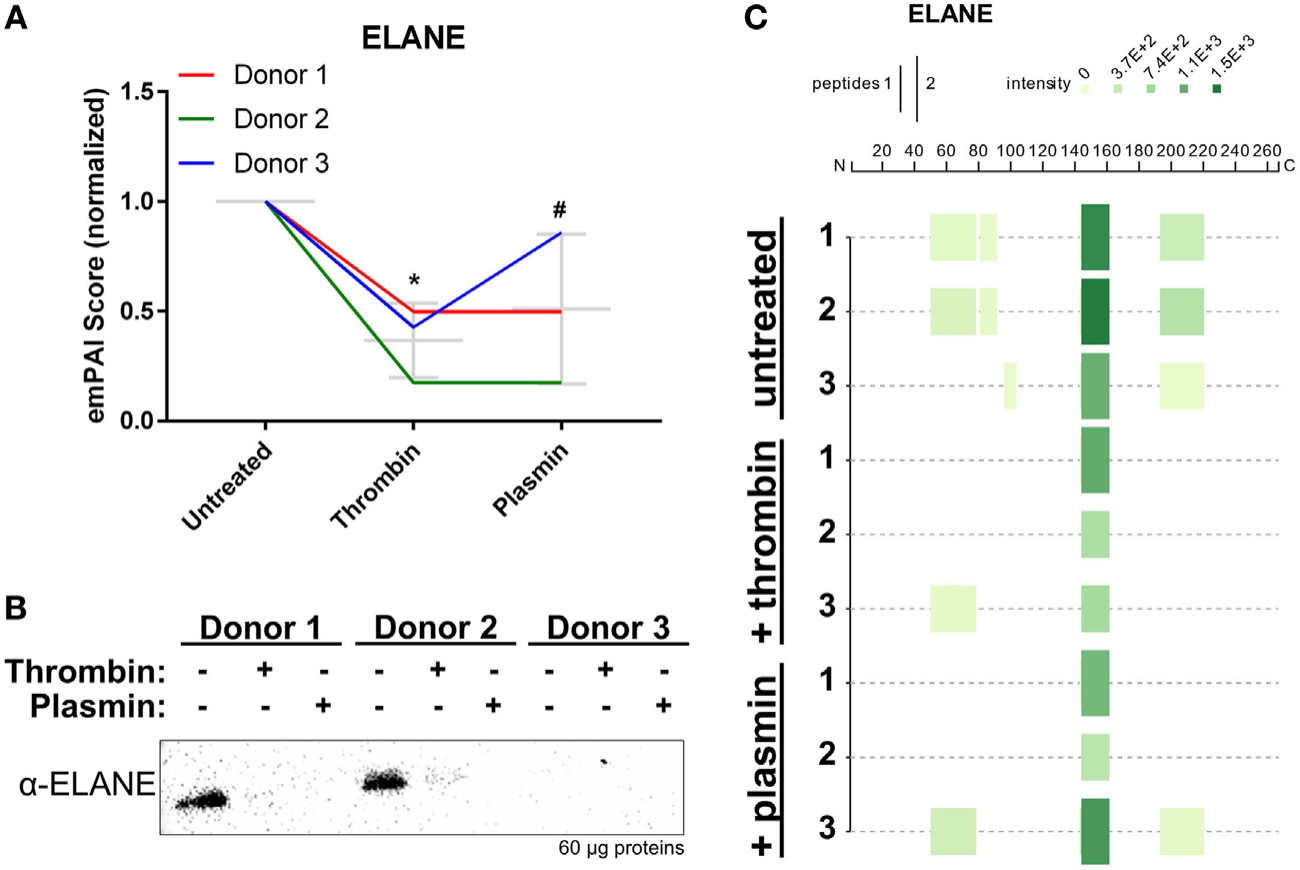
Neutrophil extracellular trap-associated neutrophil elastase is changed by thrombin and plasmin. **(A)** The ELANE’s exponentially modified protein abundance index (emPAI) scores from thrombin- and plasmin-treated samples were normalized against the respective donor’s untreated control. Donor-matched samples are presented (*n* = 3). **(B)** Western blot analysis for ELANE (α-ELANE, ~27 kDa) with 60 µg of total protein. **(C)** Peptigram for ELANE was generated using the sum of its respective peptide scores.

Mass spectrometry data showed that ELANE was significantly reduced in all three NET samples after treatment, particularly with thrombin (Figure 6A). Correspondingly, western blot analysis using polyclonal antibodies against ELANE showed that it was reduced on NETs following treatments with the proteases (Figure 6B). In agreement with the observed ELANE reduced levels, four tryptic peptides of ELANE were not detected on the thrombin- or plasmin-treated NETs from at least two donors (Figure 6C; Table S1 in Supplementary Material). Two of these four peptides were 51G→78R and 82V→91R, which lie within the first 100 residues used to generate the polyclonal antibody against ELANE. These results may explain the observation that ELANE staining was significantly reduced by the two proteases as detected by western blotting (Figure 6B).

## Discussion

Here, we report for the first time that exogenous proteases such as thrombin and plasmin can alter the proteome of NETs. In addition, we were able to identify a higher number of NET-associated proteins than previously reported. We also provide evidence on the dynamic regulation of NET-proteins in the presence of thrombin and plasmin, thus providing a conceptual base for future studies on the complex interactions between neutrophils, NETs, and the microenvironment.

Studies on the proteome of NETs have used nucleases at varying concentrations (5–20 U/ml) and incubation durations (10–40 min) (6, 7, 20, 21). Here, we used 20 U/ml DNase I for 1 h for more extensive digestion of NETs. To ensure that we accurately identify NET-bound proteins with high confidence, emPAI values of proteins detected in the non-DNase I-treated samples were subtracted from DNase I-treated samples (Figure S1 in Supplementary Material). As a result, we identified 164 NET-associated proteins, several previously not described (6, 7). However, we did not identify calprotectin S100A12 and the cathelicidin LL37 which were previously reported to bind to NETs (6, 7, 22, 23), although the corresponding peptides were detected in the LC-MS/MS spectra but were later excluded for analysis as they fell within the FDR ≤ 1. Nonetheless, we were able to identify all other proteins, even those identified by either Urban et al. (7) or O’Donoghue et al. (6) only, such as histone H1 and catalase, respectively (Table 1). Moreover, we were able to identify additional proteins, such as the protease inhibitors SerpinA1 and SerpinB1 which are expressed by neutrophils (24, 25) and can inhibit thrombin, ELANE, cathepsin G, and other serine proteases (26, 27). Indeed, SerpinB1 has been implicated in NET formation and was also recently identified on NETs (20, 28). Similarly, lipocalin-2, eosinophil cationic protein, and complement C3 were identified in our NET preparations and have been reported to be expressed by neutrophils too (29–31). The possibility that the prolonged DNase I incubation time led to release of additional proteins cannot be excluded; however, the incorporation of non-DNase I-treated controls should mitigate this possibility. Nonetheless, this shows that by employing sensitive methods and stringent protein exclusion workflow, we were able to identify previously undisclosed NET-proteins with good reproducibility.

While thrombin and plasmin were reported to interact with short single-stranded DNA (17, 18), their interaction with cell-free DNA remains unclear. Here, we show that in particular thrombin binds to NETs and/or DNA (Figure 3). Thrombin possesses two distinctive electropositive surface regions—the fibrinogen-recognition exosite I and heparin-binding exosite II (32)—which have been shown to interact with DNA aptamers (17, 33). Hence, it is likely that thrombin binds full-length DNA through these two exosites, which is of relevance as DNA of NETs was shown to promote thrombin generation in platelet poor plasma (34). We also show that plasmin can bind to DNA, albeit at higher concentrations than thrombin (Figure 3D), suggesting that the serine proteases’ DNA-binding affinities are different. Considering the early stages of wounding, the presence of cell-free DNA or NETs may therefore facilitate thrombin generation and their subsequent binding to NETs, which is logical from a physiological perspective as it compartmentalizes and thus controls thrombin activity, possibly to avoid uncontrolled fibrin generation at inflammatory sites. Conversely, the weak interaction between DNA or NETs and plasmin (or plasminogen) would ensure this enzyme is more “freely” distributed, enabling proper fibrinolysis during wounding and inflammation. In this respect, it is interesting to note that thrombin and plasmin, like ELANE, belongs to the vast family of S1 peptidases, sharing an overall similar structure and folding (35). Although our results provide evidence that particularly thrombin binds to NETs, studies on its DNA interaction domains, binding sequences, and affinities are mandated to characterize this interaction in detail. These studies are, however, outside the scope of the present study.

In addition, we show that thrombin and plasmin changed the overall NETome (Figure 2C). The treated samples shared large overall similarities in terms of the proteins identified (145 of 164 proteins; 88.4%). Of interest is the *changes in abundance* of these NET-associated proteins (Figure 4A). We noticed that majority of these classical NET-associated proteins were reduced when thrombin or plasmin was present (*cluster C3* and *C5*; Figure S4A in Supplementary Material). For example, various NET-associated histones seemed to be reduced by both thrombin and plasmin, compatible with the results showing that both thrombin and plasmin degrade histones (Figures 4B–E). On a separate note, it was also interesting that the H4 peptide (V61→K78) was missing in the thrombin- and plasmin-treated NETs, which may indicate that the proteases targeted this region (Figure 5C).

Formation of NETs can be triggered by various stimuli, including chemical inducers such as PMA and calcium ionophores, but also bacteria, fungi, and activated platelets (1, 7, 21, 36–40). Therefore, to focus on the concept that proteases may *influence* the proteome of *pre-formed* NETs, we selected PMA as the standard NET-inducing agent. Whether thrombin or plasmin induces NETosis was not addressed in this study. In this context, it is worth mentioning that it has been observed that thrombin can induce neutrophil chemotaxis and aggregation at submicromolar concentrations, although the chemotactic effect was not observed at 1 µM of thrombin (41). Moreover, plasmin has been shown to cause neutrophil aggregation and adhesion to endothelial surface *in vitro* at sub-micromolar concentrations (42, 43), albeit conflicting evidence exist concerning its adhesion-inducing effects (44). In addition, Ryan et al. (42) did not observe neutrophil lysis after treatment with plasmin (42). Although outside the scope of this study, further studies are warranted to study the proteases’ possible effects *per se* on NET formation.

The reduced histone levels on NETs may affect the overall function of NETs. Histones are antimicrobial (45) and contribute to NET-mediated antimicrobial activity (1); hence, their reduction on NETs may decrease NET-mediated antimicrobial effects. Simultaneously, levels of other NET-associated antimicrobial proteins such as defensins and azurocidin were also modulated (Figure S4A in Supplementary Material), potentially affecting the antimicrobial effects of NETs. NET-bound or released histones are cytotoxic. Apart from cellular effects, DNA and/or histones have also been reported to promote thrombin generation and cause thrombosis *in vivo*, in a platelet-independent or -dependent manner (34, 46, 47). Furthermore, histones can stabilize clots by increasing fibrin’s resistance to fibrinolysis (48). Therefore, these evidences imply that exogenous proteases, such as thrombin or plasmin, can modulate NET effects in various physiological contexts.

Interestingly, NET-associated ELANE was also reduced in the presence of thrombin and plasmin, and several tryptic peptides, present in the untreated samples, were not detected after subjection to the two proteases (Figure 6; Table S1 in Supplementary Material). Notably, two tryptic peptides—G51→R78 and Q194→R220—occupying two of the three active sites of ELANE were missing from at least two donors’ samples (Figure 6C; Table S1 in Supplementary Material). Intriguingly, It has been reported that murine ELANE auto-proteolyzes a conserved region near its S1 pocket (49), which corresponds to the peptide Q194→R220 occupying the S1 pocket of human ELANE (50). Hence, although speculative, it is possible that thrombin and plasmin displaces ELANE from NETs thus allowing ELANE to undergo auto-proteolysis, alternatively it is directly proteolyzed by thrombin and plasmin, as observed for the histones. However, the involvement of other regulatory mechanisms following thrombin and plasmin addition resulting in the observed reduction of NET-associated proteins (i.e., ELANE and histones) cannot be ruled out and require further investigations.

There is a growing interest in studying the NETome under different inflammatory conditions. A recent study on NETs induced by monosodium urate crystals show that the process of NETosis is ROS independent and that NETs are coated with actin, which protects them from nuclease degradation (20). Conversely, NETosis induced by different strains of *Pseudomonas aeruginosa* was shown to be ROS dependent and lead to generation of NET-proteins similar to those reported previously (bacterial proteins were, however, excluded from analysis) (21). Taken together, these reports, as well as our findings, illustrate that the NETome is a dynamic scaffold and further studies are merited to elucidate its temporal and qualitative changes during inflammatory-infective states. Knowledge of the dynamic changes of NETome components induced by different stimuli of relevance for different pathological states could possibly be exploited in the future search for new disease biomarkers.

## Ethics Statement

Human whole blood samples were obtained from participating subjects who gave written informed consent. The process of blood sample collection and methods used thereafter were performed in accordance with the guidelines and regulation recommended and approved by the Nanyang Technological University Singapore’s Institutional Review Board (IRB-2014-10-041).

## Author Contributions

CL, SA, and AS participated in the planning, design and interpretation of experiments, results, and validation strategies. CL prepared the NETs samples and SA further processed them for LC-MS/MS analysis. SS provided critical inputs for the LC-MS/MS data analysis and the usage of data for further analysis. CL performed the western blots and *in silico* analyses for the tryptic peptides detected and identified. YC did the EMSA experiments. RS contributed to parts on thrombin and plasmin interactions with DNA. CL, SA, and AS wrote and reviewed the manuscript. All the authors reviewed the manuscript.

## Conflict of Interest Statement

The authors declare that the research was conducted in the absence of any commercial or financial relationships that could be construed as a potential conflict of interest.
