# Supplementary material for: Thrombin and Plasmin Alter the Proteome of Neutrophil Extracellular Traps
Source: Front Immunol. 2018 Jul 9;9:1554. doi: 10.3389/fimmu.2018.01554 (PMC6046383; doi:10.3389/fimmu.2018.01554)
Supplement: Supplementary file 1 [file data_sheet_1.PDF]

Supplementary Material

Thrombin and plasmin alter the proteome of neutrophil extracellular traps

Chun Hwee Lim[1,2*], Sunil Adav[2,3], Siu Kwan Sze[3], Yeu Khai Choong[2], Rathi Saravanan[2], and Artur Schmidtchen[2,4]

*** Correspondence:** Chun Hwee Lim: limc0186@e.ntu.edu.sg

1 Supplementary Figures

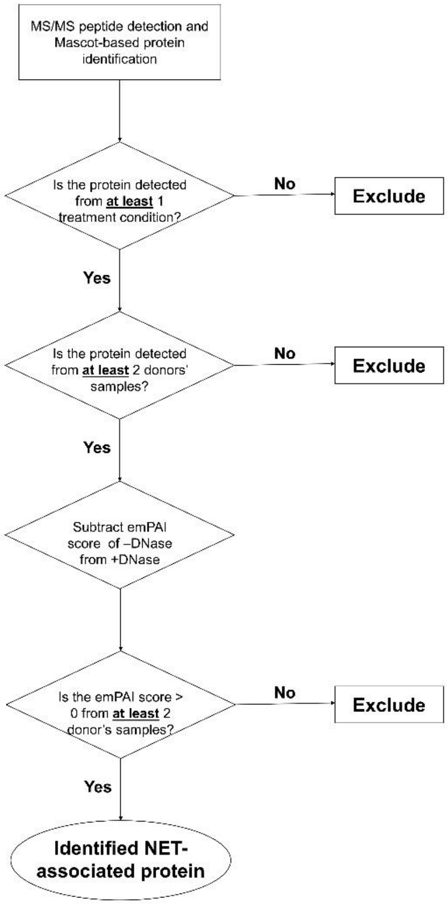

Supplementary Figure 1: Data processing workflow for the identification of NET-associated proteins. Peptides spectra were subjected to Mascot search with the specified criteria and FDR for identification. The identified proteins were first assessed for their presence in at least 1 treatment condition (i.e. untreated, thrombin- or plasmin-treated), followed by whether they were identified in at least 2 donors within the same treatment condition. This initial screening further rules out proteins that might have been non-specifically identified. Then, emPAI values of -DNase samples were subtracted

from +DNase samples to further filter out proteins that might have been constitutively released from cell membrane / debris or wells during DNase I treatment. If the emPAI score is equal or less than 0, the protein is not considered as NET-bound. Positive emPAI scores (>0) from at least 2 donors were then considered as NET-associated and used for further analyses.

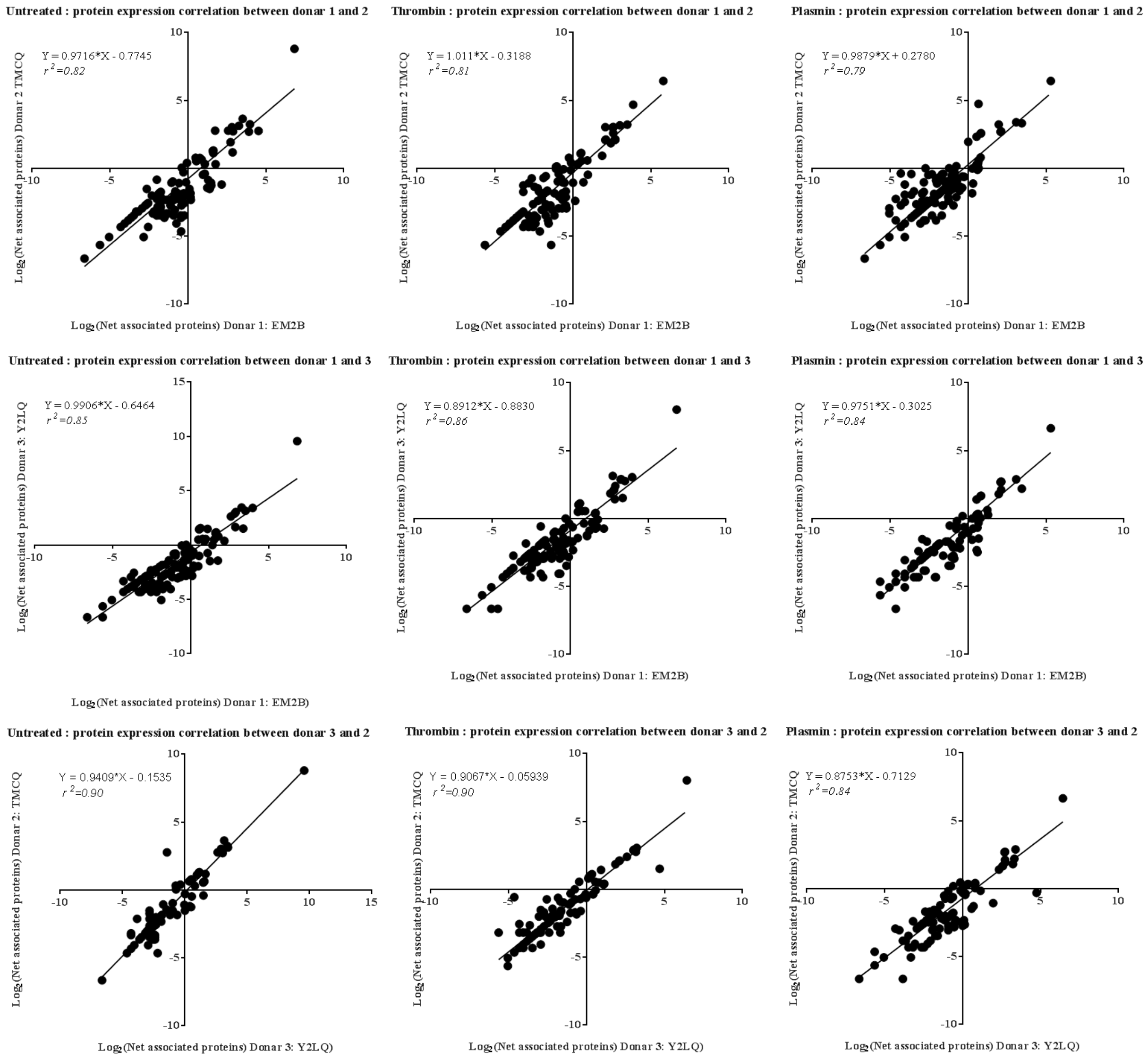

Supplementary Figure 2: Correlation analysis plots for reproducibility assessment. Linear correlation plots were generated when comparing data between the 3 donors and their respective treatments – untreated (*left column*), thrombin-treated (*middle column*) and plasmin-treated (*right column*). Pearson's correlation coefficient (r^2) was applied.

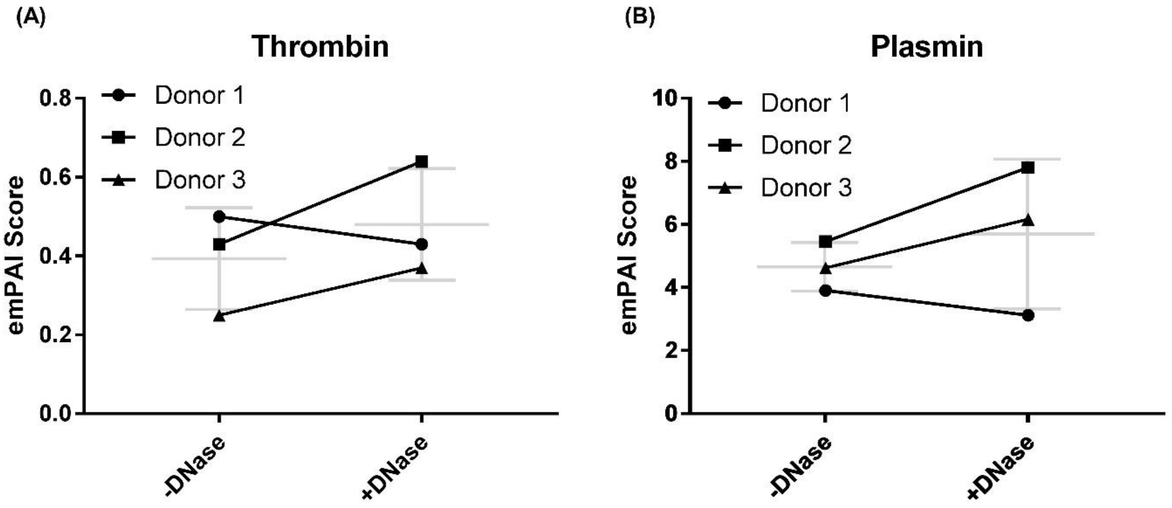

Supplementary Figure 3: Comparison between +DNase and -DNase samples. The emPAI scores for +DNase and -DNase samples of **(A)** thrombin-treated and **(B)** plasmin-treated samples were plotted to show that there was a general increase in detection following DNase I treatments.

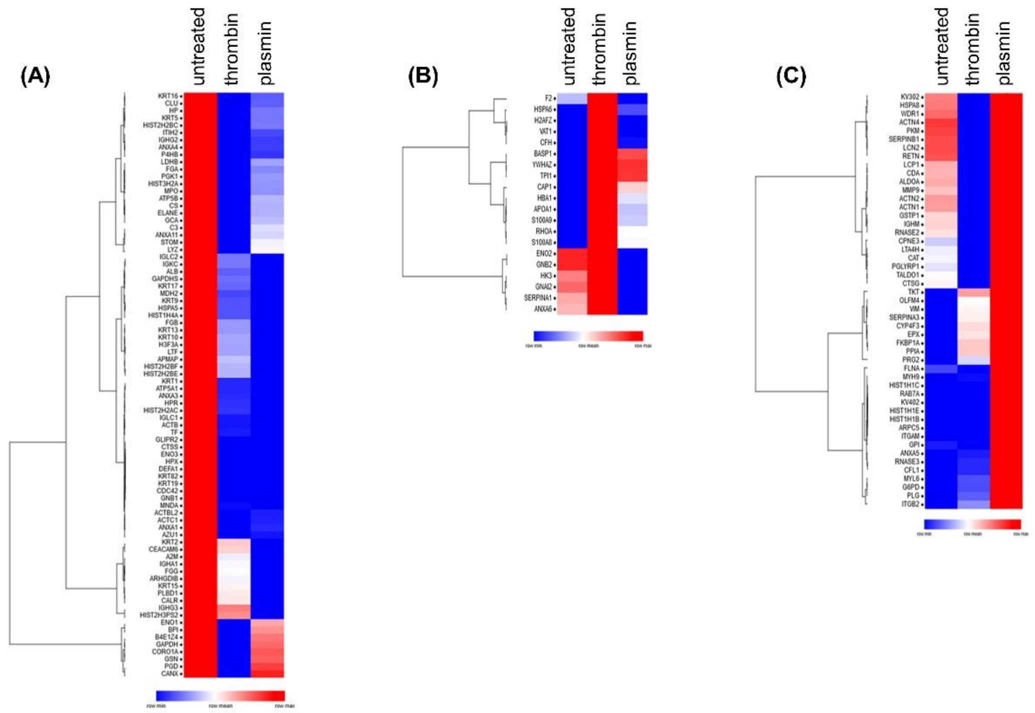

Supplementary Figure 4: hierarchical clusters of NET-associated proteins. Segregated view of the hierarchical clusters – **(A)** proteins enriched in untreated samples (*cluster C3* and *C5*), **(B)** proteins enriched in thrombin-treated samples (*cluster C1, C2 and C4*) and **(C)** proteins enriched in plasmin-samples (*cluster C6*). Enriched proteins are displayed in *red*, lowered proteins are displayed in *blue* and the intermediate values are displayed in the shades of *red* and *blue*.

2 Supplementary Table

Supplementary Table 1: Tryptic peptides of neutrophil elastase (ELANE). Summed peptide scores for each peptide from the respective samples are presented. The value = 0 denotes that the peptide was not identified.

			Untreated			Thrombin			Plasmin		
**Peptide Sequences**	**Peptide Start**	**Peptide End**	**Donor 1**	**Donor 2**	**Donor 3**	**Donor 1**	**Donor 2**	**Donor 3**	**Donor 1**	**Donor 2**	**Donor 3**
GGHFCGATLIAPNFVMSAAHCVANVNVR	51	78	87.6	181.73	**0**	**0**	**0**	69.53	**0**	**0**	287.36
VVLGAHNLSR	82	91	50.91	78.18	**0**	**0**	**0**	**0**	**0**	**0**	**0**
QVFAVQR	97	103	**0**	**0**	32.9	**0**	**0**	**0**	**0**	**0**	**0**
RLGNGVQCLAMGWGLLGR	144	161	117.91	264	155.46	128.02	**0**	**0**	68.37	**0**	378.99
LGNGVQCLAMGWGLLGR	145	161	1255.71	1212.97	915.14	981.59	624.88	690.72	932.92	489.53	880.71
QAGVCFGDSGSPLVCNGLIHGIASFVR	194	220	321.14	552.33	27.25	**0**	**0**	**0**	**0**	**0**	77.31
